# Surgical treatment of infective endocarditis in intravenous drug abusers

**DOI:** 10.1186/s13019-021-01491-1

**Published:** 2021-04-20

**Authors:** Alina Zubarevich, Marcin Szczechowicz, Anja Osswald, Jerry Easo, Arian Arjomandi Rad, Robert Vardanyan, Bastian Schmack, Arjang Ruhparwar, Konstantin Zhigalov, Alexander Weymann

**Affiliations:** 1 Department of Thoracic and Cardiovascular Surgery, West German Heart and Vascular Center, University of Duisburg-Essen, Essen, Germany; 2 Department of Medicine, Faculty of Medicine, Imperial College London, London, UK

**Keywords:** Infective endocarditis, Intravenous drug abuse, High risk valve procedures

## Abstract

**Background:**

Despite current progress in antibiotic therapy and medical management, infective endocarditis remains a serious condition presenting with high mortality rates. It also is a life-threatening complication in patients with a history of chronic intravenous drug abuse. In this study, we analyzed our institutional experience on the surgical therapy of infective endocarditis in patients with active intravenous drug abuse. The aim of the study is to identify the predictive factors of mortality and morbidity in this subgroup of patients.

**Methods:**

Between 2007 and 2020, a total of 24 patients (7 female, mean age 38.5 ± 8.7) presenting with active intravenous drug abuse underwent a surgical treatment for the infective endocarditis at out center. The primary endpoint was survival at 30th day after the surgery. The secondary composite endpoint included freedom from death, recurrent endocarditis, re-do surgery, and postoperative stroke during the follow-up period. Mean follow-up was 4.2 ± 4.3 years.

**Results:**

Staphylococcus species was the most common pathogen detected in the preoperative blood cultures. Infection caused by Enterococcus species as well as liver function impairment were identified as mortality predictor factors. Logistic EuroSCORE and EusoSCORE-II were also predictive factors for mortality in univariate analysis. Survival at 1 and 3 years was 78 and 72% respectively. Thirty-day survival was 88%. 30-day freedom from combined endpoint was 83% and after 1 and 3 years, 69 and 58% of the patients respectively were free from combined endpoint. Five patients (20.8%) were readmitted with recurrent infective endocarditis.

**Conclusion:**

In patients presenting with active intravenous drug abuse, treatment of infective endocarditis should be performed as aggressively as possible and should be followed by antibiotic therapy to avoid high mortality rates and recurrent endocarditis. Early intervention is advisable in patients with an infective endocarditis and enterococcus species in the preoperative blood cultures, liver function deterioration as well as cardiac function impairment. Attention should be also payed to addiction treatment, due to the elevated relapse rate in patients who actively inject drugs. However, larger prospective studies are necessary to support our results. As septic shock is the most frequent cause of death, new treatment options, e.g. blood purification should be evaluated.

## Background

Despite numerous anti-drug initiatives, the global rate of intravenous drug abuse has been sharply rising, with an estimated 15.6 million worldwide users in 2015 [1].

Intravenous drug abusers are at an increased risk of infective endocarditis (IE), due to the bacteremia caused by unsterile injections. Their population is mostly constituted by relatively healthy young adults with no underlying cardiac diseases. Nevertheless, despite the patients’ young age, IE presents itself as a challenging condition with high mortality rates. Overall, even in the era of modern medicine with targeted antibiotic therapy IE in patients with active intravenous drug use (IVDU) presents with 1-year mortality rates of up to 40% [2]. Approximately 60 to 70% of cases require cardiac surgery, which carries an extremely high risk due to the often critical risk posed by sepsis or congestive heart failure. Furthermore, the rate of recurrent IE remains high. Additionally, most cases of IE in IVDU are due to multiple antibiotic resistant and highly virulent organisms, thus having a negative influence on the effectiveness of antibiotics and leading to IE recurrences [2, 3]. The surgeons’ eagerness to perform surgeries on patients with active IVDU is cooled down due to the noncompliance of the patients and high rates of relapsing on the drug use. Unfortunately, when being provided surgical and medical therapy to prevent recurrence of IE, not all of IVDU patients receive appropriate addiction treatment and psychiatric counselling, especially those patients who are actively injecting.

In this study we sought to review our institutional experience with the surgical treatment of patients with IE and active IVDU, to contribute to the limited number of studies available on this issue to identify the predictive factors of mortality and to review our strategies of care in this particular patient cohort.

## Methods

### Study design and patient cohort

Between 2007 and 2020, a total of 24 patients (7 female, mean age 38.5 ± 8.7) presenting with intravenous drug use (IVUD) underwent surgical treatment for infective endocarditis (IE) at our center. The patients enrolled received a diagnosis of IE according to the modified Duke criteria [4] and were current intravenous drug users until the hospital admission. Data collected prospectively as a part of the institutional database included detailed information on patients’ demographics, baseline clinical characteristics, their laboratory tests, preoperative medical history, echocardiographic and hemodynamic parameters, as well as intraoperative variables and postoperative outcomes. The study was approved by the local ethic committee. All patients signed the informed consent on follow-up. Follow-up was performed via telephone interview with the patients’ GPs and/or the patients. The data on mortality was provided by the local city hall bureau of vital statistics if the information was unobtainable from the medical records.

The primary endpoint in this study was survival at 30th day after the surgery. The secondary composite endpoint included freedom from death, recurrent endocarditis, re-do surgery, and postoperative stroke during the follow-up period. The aim of the study was to evaluate our institutional experience of IE in IVDUs and to identify factors influencing morbidity and mortality in this cohort.

### Diagnostic criteria and indications for surgical treatment

The diagnosis of IE was made using a combination of laboratory tests, clinical findings and the information acquired from transesophageal echocardiography, according to the modified Duke criteria. During preoperative work-up, all the patients underwent abdominal ultrasound and a full body CT-scan to obtain information about the septic foci. Absolute indications for the urgent of emergent procedure were: prosthetic valve endocarditis, progressive heart failure, unmanageable infection despite optimal antibiotics, septic embolism and acute renal failure. Each patient in our study matched at least one of those criteria.

### Surgical technique

All procedures were performed on cardiopulmonary bypass with mild hypothermia. Myocardial protection was achieved with antegrade cold crystalloid cardioplegic solution. Mitral- and tricuspid valve procedures were performed with occlusion of both caval veins. Mitral- and aortic valve procedures were performed on the arrested heart and isolated tricuspid valve procedures were performed either in cardiac arrest or on beating heart according to surgeons’ preferences.

The treatment concept of the IE in our institution implies radical debridement of the infected tissue followed by irrigation with antibiotic solution. All visibly infected and necrotic structures were resected and all the abscesses were evacuated and if needed were filled with gentamicin-glue and covered with bovine pericardial patch (St. Jude Medical, Saint Paul, Minnesota, USA). Valve repair was preferred over valve replacement if enough of the valve anatomy was intact after the debridement. Defects were closed with pericardial patches or if possible, by direct stitches. The use of the foreign materials was avoided when possible.

In cases of valve replacement, the type of chosen prosthesis was generally determined by each surgeons’ preference together with patients’ wish. Prosthesis, sutures and patch material were soaked in gentamicin solution before use.

### Follow-up

Follow-up was performed either by telephone interview with the patients’ general practitioners who also performed the postoperative echocardiography, or by telephone contact with patients and/or family members. The data on mortality was provided by the local city hall’ bureau of vital statistics if the information was unobtainable from the medical records.

### Postoperative care

An important goal of postoperative therapy was controlling the local and systemic inflammatory process. In general, broad spectrum antibiotic therapy was initially started which was then directed against the infecting microorganisms. If there was a rapid decline of the infective signs, we deescalated the therapy. If no microorganisms were detected, a broad calculated triple therapy with imipenem and vancomycin was applied. In every case, intravenous antibiotic therapy was continued for 6 weeks postoperatively.

Transesophageal and transthoracic echocardiography were performed regularly over the course of treatment to exclude signs of the recurrent vegetations or paravalvular leakage. The latter, in combination with persisting signs of infection would constitute an indication for reoperation due to the strong suspicion of a recurrent IE.

### Statistical analysis

Surgical treatment technique was not randomized, it was rather determined by the best medical judgment based on each individual case. Data was collected from chart reviews. Statistical analysis was performed using IBM SPSS version 26 (IBM Corp., Chicago, Ill., USA) and R software v.3.4.3 (R Foundation for Statistical Computing, Vienna, Austria). The data was checked for normality using the Shapiro-Wilk test. If data was not normally distributed, continuous variables are expressed as the medians (interquartile range, IQR). Categorical variables are expressed as frequencies and percentages. We used the Kaplan-Meier method to analyze survival. The significance of survival differences between the groups was assessed with Log-Rank and Breslow tests. A value of *p* < 0.05 was considered to be statistically significant.

### Definitions

Septic shock - a subset of sepsis in which particularly profound circulatory, cellular, and metabolic abnormalities [5]. Acute kidney failure on dialysis - a rapid fall in the rate of glomerular filtration, which manifests clinically as an abrupt and sustained increase in the serum levels of urea and creatinine with an associated disruption of salt and water homeostasis treated on dialysis [6].

## Results

**Fig. 1:**
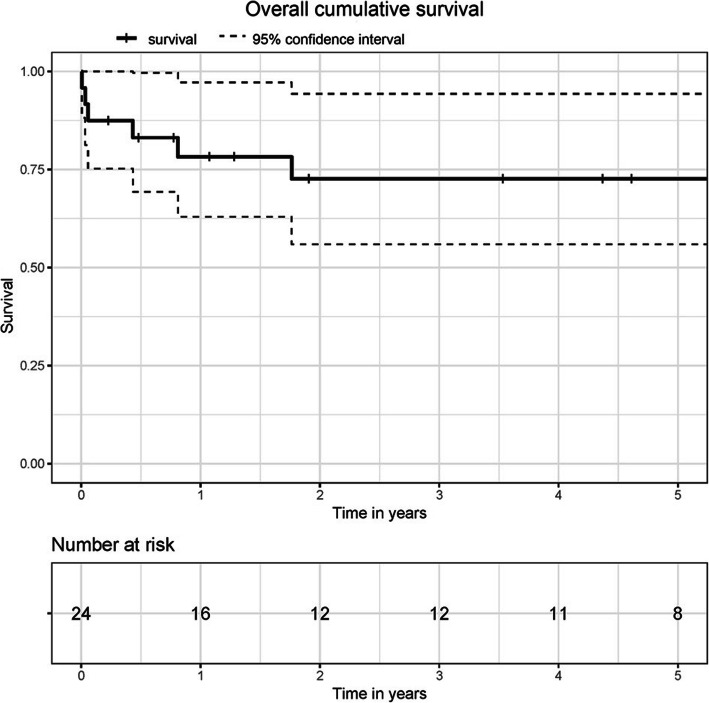
Cumulative survival

**Fig. 2:**
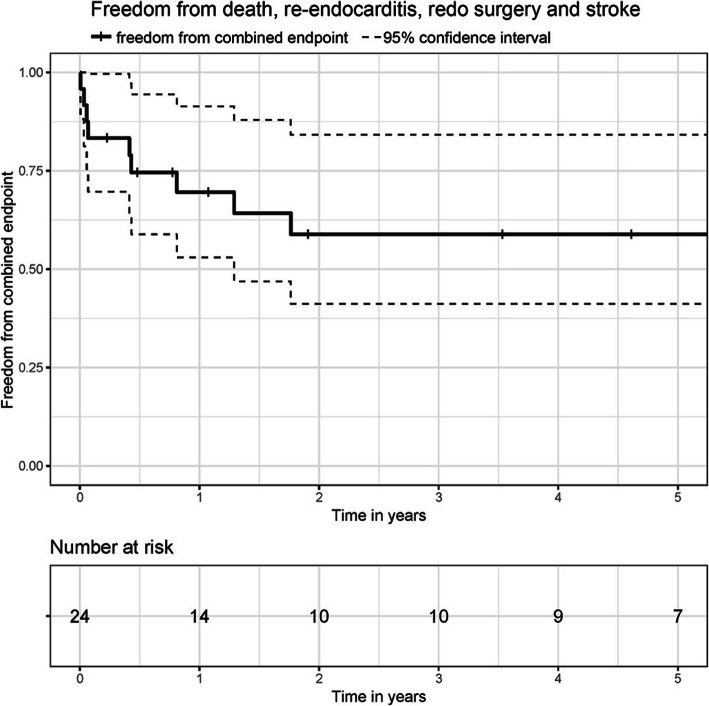
Freedom from death, re-endocarditis, redo surgery and stroke

The mean age of the cohort was 38.5 ± 8.7 years with 29.2% of the patients being female (Table 1). All patients in the study were active intravenous drug users at the moment of admission. Previously, one of the patients underwent a tricuspid valve replacement procedure due to tricuspid valve endocarditis. Further medical history included Hepatitis C (79.2%), kidney injury (29.2%), liver cirrhosis (16.7%) and HIV (12.5%). Blood cultures were positive in 70.8% of the patients with *Staphylococcus aureus* identified in 82.4% of them. Infection focus was unknown in 70.8% of the patients. Prior to the admission, 62.5% of the cohort suffered septic embolism. The mean CRP level and leukocytes count were 6.5 ± 5.5 mg/dl and 13.6 ± 11.3 mg/dl respectively. Median logistic EuroSCORE was 15.3% (CI 9.3–18.8) and EuroSCORE II 4.84% (CI 2.9–7.9) (Table 2). The most affected valve was the tricuspid valve (62.5%, *n* = 15). Four patients (16.7%) underwent a multivalve procedure (Table 3). Overall cumulative survival at 30 days, 1 year and 3 years was 88, 78 and 72% respectively (Fig. 1). Freedom from the combined events at secondary endpoint was 83% at 30 days, 68% at 1 year and 58% at 3 years (Fig. 2).

**Table 1:** Preoperative data

Female	7 (29.2%)
Age, years	38.5 ± 8,7
BMI, kg/m^2^	24,7 ± 4.3
NYHA-Class	2.7 ± 0.6
Chronic kidney injury	7 (29.2%)
Liver cirrhosis	4 (16.7%)
Hepatitis C	19 (79.2%)
HIV	3 (12.5%)
Positive blood cultures	17 (70.8%)
*Staphylococcus aureus*	14 (58.3%)
*Staphylococcus epidermidis*	1 (4.2%)
Enterokokkus spiecies	2 (8.3%)
Leukocytes, mg/dl	13.55 ± 11.3
CRP, mg/dl	6.5 ± 5.5
Septic emlolism	15 (62.5%)
EuroSCORE II, %	4.84 (2.9–7.9)
logistic EuroSCORE I, %	15,3 (9.31–18.8)

*BMI* Body Mass Index, *CRP* C-reactive Protein, *HIV* Human Immunodeficiency Virus, *NYHA* New York Heart Association

**Table 2:** Intraoperative data

Bypass time, min	95.33 ± 51.1
Operating time, min	178.4 ± 61.4
Cross-clamp time, min	49.13 ± 39.8
Lateral thoracotomy	2 (8.3%)
*Type of valve surgery*	
Mechanical prosthesis	1 (4.2%)
Biological prosthesis	16 (66.7%)
Valve repair	7 (29.1%)
*Site of valve surgery*	
Aortic	6 (25%)
Mitral	7 (29.1%)
Tricuspid	15 (62.5%)
Aortic+ mitral valve	3 (12.5%)
Aotric+mitral+tricuspidal	1 (4.2%)
Multivalve surgery	4 (16.7%)
Elective	3 (12.5%)
Urgent	15 (62.5%)
Emergent	6 (25%)

**Table 3:** Postoperative outcomes

Atrial fibrillation	4 (16.7%)
AVB III	4 (16.7%)
Permanent pacemaker	2 (8.3%)
Acute kidney failure on dialysis	8 (33.3%)
Coagulation disorder	3 (12.5%)
Reopening for bleeding	2 (8.3%)
Cerebrovascular accident	0
Recurrent IE	5 (20.8%)
Re-operation because of IE	4 (16.7%)
Deep sternal wound infection	1 (4.2%)
Septic shock	3 (12.5%)
Multiorgan failure	4 (16.7%)
Follow-up time, years	4.2 ± 4.3
In-hospital mortality	3 (12.5%)
30-day-mortality	3 (12.5%)

*AVB* Atrioventricular block, *IE* Infective endocarditis

To evaluate independent predictors of 30-day mortality, a logistic regression model was constructed. Several univariate indicators were found to predict 30-day mortality. This model showed that logistic EuroSCORE I (odds ratio [OR], 1.078; 95% confidence interval [CI], 1.017–1.142; *P* = 0.012), EuroSCORE II (OR, 1.339; 95% CI, 1.117–1.606, *P* = 0.002), history of Liver cirrhosis (OR, 8.149; 95% CI, 1.615–41.12, (to *P* = 0.011), and positive blood cultures with Enterococcus species (OR, 0.997; 95% CI, 0.181–5.489, *P* = 0.03), were significantly associated with 30-day mortality.

## Discussion

In patients presenting with active intravenous drug use, IE is a severe condition associated with high mortality and recurrence rates [7, 8]. Even though, multiple research groups have published numerous articles on IE in IVDUs, an ongoing debate about the prognosis in this challenging pathology still persists [9]. This report describes our single center experience on the surgical treatment of IE in patients with IVDU over a period of 13 years and analyses the predictors of mortality. The aim of this study is to identify the factors which increase the mortality in this particular cohort. The results of our study could lead to improvement of surgical therapy and postoperative care.

In our cohort, overall cumulative mortality at 30 days, 1 year and 3 years was 12, 22 and 28% respectively (Fig. 1). A high 30-day mortality of 12% is rather atypical for the patients with IVDU according to the most trials conducted by other study groups [10, 11]. On the other hand, 87.5% of the patients underwent an urgent or emergent procedure, which could explain the unexpected poor short-term survival. After 1 year, the mortality rate was 22%, which is in line with the results published in the literature. The reason for low short-term mortality rates of the patients with active IVDU is the young patients’ age, few comorbidities and more common involvement of the right-sided valves [11–13]. In our study, 62.5% of patients (*n* = 15) suffered from tricuspid valve endocarditis, which correlates with a rate of right-sided valves involvement as described by Kim at al [11].

Unfortunately, due to their recurrent endocarditis, patients with IVDU suffering from IE have much higher readmission rates when compared with endocarditis patients in the general population [14–16]. Patients with IVDU present with higher mortality between postoperative days 90 and 180 [8]. In our study, readmission due to recurrent IE stands at 22.5% (*n* = 5) of patients. Four of them 16.7% underwent a further cardiac procedure for IE and one patient (4.2%) underwent a successful antibiotic treatment. All patients suffering from the recurrent IE in our cohort also started injecting drugs again.

Recent literature reports the relapse rates of intravenous drug use after hospital discharge at a range between 22 and 49% [14, 17, 18]. In our cohort, some of the patients were participating in the methadone-program, receiving daily opioid substitute from specialized centers, but at the time of admission to the hospital all of them were still active drug users. Unfortunately, additionally to the medical addiction treatment no patient was offered any psychiatric treatment or had a caring social worker at their side. In one institutional study, more than half of the patients discharged from the hospital were lacking any kind of planned addiction treatment [17]. Due to high relapse rates and the consequent high rates of recurrent IE in patients with active IVDU, the concept of medication associated treatment (MAP) should be adopted. MAP involves medical treatment, psychiatric evaluation and counseling, which should be considered in every hospital-treated IVDU patient with IE. As reported by Mohlman et al., lower mortality and morbidity rates, lower hospital cost and overall less referrals to different departments of the health system was found in MAP enrolled patients [19]. The current situation is that patients with IVDU are at greater risk of recurrent endocarditis between 3 and 6 months when compared to the patients with no history of drugs abuse, given both population are being treated with antibiotics for 6 weeks postoperatively [20]. The hazard of death after reoperation for recurrent IE in IVDU-patients is 10 times higher than in non-drug-users. Therefore, it is crucial that in this group of patients appropriate addiction therapy is offered along pathogen targeted antibiotic therapy,

Septic shock was at 12.5% (*n* = 3) the most common cause of death in our cohort. Haidari et al. described the use of the intraoperative hemadsorption in patients with native valve infective endocarditis and illustrated a significant reduction in postoperative sepsis and sepsis-associated deaths in patients who received intraoperative hemadsorption [21]. The use of blood purifying mechanisms such as hemadsorption in patients with acute IE and chronic IVDU could be a way to reduce the number of sepsis-associated postoperative deaths. Moreover, patients receiving hemadsorption seem to show better hemodynamic stability and lower rates of postoperative organ failure [22].

The most common pathogen in our cohort was *Staphylococcus aureus* (Table 1)*.* Our results match with previously published studies which reported that most patients with IVDU and IE present with *Staphylococcus aureus* infection [23–25]. Although, some research groups reported increased mortality rates in patients suffering from IE with *Staphylococcus aureus* infection [26, 27], our cohort has shown that infection with *Enterococcus faecalis* was the predictive factor for mortality in univariate analysis (Table 4). Antibiotic treatment remains an important part of the endocarditis treatment and in some cases it may be used as the sole treatment modality. In our institution we perform without exceptions a six-week intravenous antibiotic therapy tailored to the pathogen revealed in the bloodstream. There are reports of abbreviated antibiotic therapy, performed in uncomplicated cases of right sided IE with either oral or intravenous antibiotics. Unfortunately, this concept is usually not suitable for patients undergoing an urgent or emergent procedures with already impaired organ function [28].

**Table 4:** Independent risk factors of 30-day mortality – univariate logistic regression

*Characteristics*	*Odds ratio with 95%Confidence interval*	*p-value*
Female gender	0.384 (0.045 to 3.306)	0.383
Age	1.022 (0.926 to 1.127)	0.672
**Logistic EuroSCORE I**	1.078 (1.017 to 1.142)	**0.012**
**EuroSCORE II**	1.339 (1.117 to 1.606)	**0.002**
NYHA Class	1.445 (0.378 to 5.527)	0.591
Kidney disease	2.844 (0.571 to 14.166)	0.202
GFR	0.973 (0.931 to 1.017)	0.225
Creatinine	1.151 (0.299 to 4.432)	0.838
**Liver cirrhosis**	8.149 (1.615 to 41.118)	**0.011**
Bilirubin	1.890 (0.782 to 4.563)	0.157
Hepatitis C	1.456 (0.170 to 12.472)	0.732
HIV	0.040 (0.01 to 1560.464)	0.552
Large vegetation	2.160 (0.252 to 18.527)	0.483
Septic embolism	1.307 (0.239 to 7.145)	0.758
Positive blood culture	0.997 (0.181 to 5.489)	0.997
Negative blood culture	0.398 (0.046 to 3.433)	0.402
Staphylococcus species	0.337 (0.068 to 1.839)	0.209
**Enterococcus species**	7.348 (1.218 to 44.323)	**0.030**
Operation time	0.998 (0.984 to 1.012)	0.780
Cross clamp time	1.002 (0.983 to 1.021)	0.829
Multivalvular endocarditis	0.046 (0.0 to416017.507)	0.706

*HIV* Human immunodeficiency virus, *GFR* Glomerular filtration rate, *NYHA* New York Heart Association Functional Classification

There is an ongoing debate about the most precise score to predict the surgical risk in patients with acute IE. In our study, the logistic EuroSCORE and EuroSCORE II appeared to be the mortality predictors in the univariate analysis. However, some research groups are working on alternative scores to predict the operative mortality [29]. Furthermore, liver cirrhosis was an independent mortality predictor in the univariate analysis. This finding can be explained due to a small number of patients and should be definitely reevaluated on a larger population.

In our institution we aim for the radical approach while operating on patients with IE. In some cases, radical surgical debridement may cause severe damage of the conduction system and lead to the complete AV block postoperatively. In our cohort there were 4 patients presenting with AV-Bloc III postoperatively with two of them (8.3%) requiring a permanent pacemaker. Because of the relatively low number of patients, further studies on larger cohorts need to be performed to support these findings.

There is a constant contradiction on the type of the valve prosthesis to use in patients with acute IE and especially in patients with IVDU, who often lack compliance.

At our institution, we tend to repair the right-sided valves and replace the left-sided valves. The current guidelines on valve surgery recommend to go for a valve repair, if possible, also in cases of IE [30]. There are various techniques of tricuspid valve repair in patients with acute IE [31, 32]. There are contradicting evidences on the type of the valve prosthesis used in case of potential valve replacement. The current state is that there is no difference in survival or recurrence of IE after using biological or mechanical valve prosthesis [33]. In our cohort, despite of the young patients’ age, we implanted biological prostheses in 66.7% of cases to avoid anticoagulation regimen, given a questionable compliance of the patients with active IVDU. Regardless of the surgical method, many surgeons are less likely to operate on patients with IE and IVDU due to an increased risk of IE recurrence and its associated higher operative mortality and a lower success of antibiotic treatment [11].

## Conclusion

In patients presenting with active intravenous drug abuse, treatment of infective endocarditis should be performed as aggressively as possible followed by antibiotic therapy to avoid high mortality rates due to recurrent endocarditis. Early intervention is advisable in patients with an infective endocarditis and enterococcus species in the preoperative blood cultures, liver function deterioration as well as cardiac function impairment, as these factors predict 30-day mortality. Attention should be also payed to addiction treatment, due to the elevated relapse rate in patients who actively inject drugs. However, larger prospective studies are necessary to support our results. As septic shock is the most frequent cause of death, new treatment options, e.g. blood purification should be evaluated.

### Study limitations

We have to report on several study limitations. Alongside with the observational non-randomized type of study from a single center, we were limited by the number of patients. Because of the rareness of the described condition in the clinical practice, we were not able to provide a larger size of the sample, so further multicenter clinical studies have to be performed to allow for generalizability of the results. Moreover, this kind of clinical studies is strongly limited by its’ ethical unfeasibility, as a direct comparison of the surgical and medical treatment is required to analyze the results and increase the statistical power. Another important point we have to address is that the patients admitted were referred from different hospitals, therefore making it impossible to determine the onset time of the IE in each case.

## Data Availability

The datasets used and analysed during the current study are available from the corresponding author on reasonable requests.
